# Prediction of oxygen supplementation by a deep-learning model integrating clinical parameters and chest CT images in COVID-19

**DOI:** 10.1007/s11604-023-01466-3

**Published:** 2023-07-13

**Authors:** Naoko Kawata, Yuma Iwao, Yukiko Matsuura, Masaki Suzuki, Ryogo Ema, Yuki Sekiguchi, Hirotaka Sato, Akira Nishiyama, Masaru Nagayoshi, Yasuo Takiguchi, Takuji Suzuki, Hideaki Haneishi

**Affiliations:** 1 Department of Respirology, Graduate School of Medicine, Chiba University, 1-8-1, Inohana, Chuo-ku, Chiba-shi, Chiba, 260-8677 Japan; 2 Graduate School of Science and Engineering, Chiba University, Chiba, 263-8522 Japan; 3 Medical Mycology Research Center (MMRC), Chiba University, Chiba, 260-8673 Japan; 4 Center for Frontier Medical Engineering, Chiba University, 1-33, Yayoi-cho, Inage-ku, Chiba-shi, Chiba, 263-8522 Japan; 5 Institute for Quantum Medical Science, National Institutes for Quantum Science and Technology, 4-9-1, Anagawa, Inage-ku, Chiba-shi, Chiba, 263-8555 Japan; 6 Department of Respiratory Medicine, Chiba Aoba Municipal Hospital, 1273-2 Aoba-cho, Chuo-ku, Chiba-shi, Chiba, 260-0852 Japan; 7 Department of Respirology, Kashiwa Kousei General Hospital, 617 Shikoda, Kashiwa-shi, Chiba, 277-8551 Japan; 8 Department of Respirology, Eastern Chiba Medical Center, 3-6-2, Okayamadai, Togane-shi, Chiba, 283-8686 Japan; 9 Department of Radiology, Soka Municipal Hospital, 2-21-1, Souka, Souka-shi, Saitama, 340-8560 Japan; 10 Department of Radiology, Chiba University Hospital, 1-8-1, Inohana, Chuo-ku, Chiba-shi, Chiba, 260-8677 Japan

**Keywords:** COVID-19, Disease severity prediction, Chest CT images, Deep learning, Explainable AI

## Abstract

**Purpose:**

As of March 2023, the number of patients with COVID-19 worldwide is declining, but the early diagnosis of patients requiring inpatient treatment and the appropriate allocation of limited healthcare resources remain unresolved issues. In this study we constructed a deep-learning (DL) model to predict the need for oxygen supplementation using clinical information and chest CT images of patients with COVID-19.

**Materials and methods:**

We retrospectively enrolled 738 patients with COVID-19 for whom clinical information (patient background, clinical symptoms, and blood test findings) was available and chest CT imaging was performed. The initial data set was divided into 591 training and 147 evaluation data. We developed a DL model that predicted oxygen supplementation by integrating clinical information and CT images. The model was validated at two other facilities (n = 191 and n = 230). In addition, the importance of clinical information for prediction was assessed.

**Results:**

The proposed DL model showed an area under the curve (AUC) of 89.9% for predicting oxygen supplementation. Validation from the two other facilities showed an AUC > 80%. With respect to interpretation of the model, the contribution of dyspnea and the lactate dehydrogenase level was higher in the model.

**Conclusions:**

The DL model integrating clinical information and chest CT images had high predictive accuracy. DL-based prediction of disease severity might be helpful in the clinical management of patients with COVID-19.

**Supplementary Information:**

The online version contains supplementary material available at 10.1007/s11604-023-01466-3.

## Introduction

The pandemic of the coronavirus disease 2019 (COVID-19) has caused > 750 million infections and 6.8 million deaths worldwide as of 1 March 2023 [1]. The overall trend in infections is downward, but there are still no signs of disease eradication. Rapid and effective triage also remains an unresolved issue for optimal treatment and effective allocation of limited healthcare resources [2].

The viral nucleic acid real-time reverse transcriptase chain reaction (RT-PCR) is the gold standard for the diagnosis of COVID-19 infection [3, 4]. RT-PCR has several limitations, however, such as dependence of the diagnosis on the viral load and sampling technique [5]. With the rapid increase in the number of cases, there are problems regarding the time required for testing and the lack of reagents. Furthermore, challenges remain, including the diagnosis of severe acute respiratory syndrome coronavirus 2 (SARS-CoV-2) pneumonia and determination of disease severity [6].

Numerous studies have shown the utility of chest CT images for the diagnosis of SARS-CoV-2 pneumonia [7–9], even in PCR-negative cases [10]. SARS-CoV-2 pneumonia is characterized by ground glass opacities in the lung parenchyma bilaterally on radiographs [11]; subsequently, infiltrating shadows are apparent. Numerous reports have quantified ground glass opacities, semi-consolidated, and consolidated lesions, and concluded that abnormal lung areas exceeding a specified value are associated with severe disease [12, 13]. Analyses using radiomic features have also been reported [14].

The relationship between COVID-19 disease severity and clinical characteristics has also been reported. Early reports have described the patient characteristics that are associated with severe disease, including older age, gender, obesity, and co-morbidities, such as hypertension, diabetes, chronic lung diseases and coronary artery disease [15–17]. Subsequent reports indicated that coagulopathies and vasculitis contribute to critical illness in patients with COVID-19 [18, 19]. Markers of lung fibrosis, such as sialylated carbohydrate antigen (KL-6), have also been reported to contribute to the progression from pneumonia to secondary lung fibrosis [20].

In parallel, deep learning (DL)-based chest image analysis has been used to predict COVID-19 disease severity, survival, and death [21], with some reports showing a diagnostic accuracy > 80% [22]. A recent study proposed nomograms and scoring systems using DL to determine COVID-19 patient status and predict critical illness [23]. However, a DL model has not been fully described in detail using clinical information and chest images together [24]. It has been recently reported that a DL model using x-rays predicts the presence or absence of oxygen supplementation, a predictor of hospitalization and delayed discharge, which is associated with disease severity [25]. Thus, we considered a DL model that combined clinical and CT imaging findings to predict oxygen supplementation in an early stage. In addition, if we enable visualization of the elements on which the DL model is built, such a DL model will facilitate healthcare professionals’ efforts to provide appropriate treatment and allocate healthcare resources in the next pandemic and for other respiratory diseases.

Therefore, we constructed a DL model for predicting oxygen supplementation at an early stage in COVID-19 infection that integrated clinical information and chest CT images.

## Methods

### Study subjects

To construct the prediction model, we enrolled 819 consecutive COVID-19 patients who were hospitalized and treated at Chiba Aoba Municipal Hospital (No. 20200301) Municipal Hospital from February 2020 to September 2021. The subjects were required to meet all of the following inclusion criteria: (1) Patients with symptoms suspicious for COVID-19 who were diagnosed with COVID-19 during the COVID-19 outbreak; (2) Patients who underwent RT-PCR tests of nasopharyngeal swab samples to establish a COVID-19 diagnosis; and (3) Patients with a positive PCR test result and a request for treatment and hospitalization from the local health department. We excluded subjects under 20 years of age (n = 31), subjects who did not undergo CT scanning (n = 32), data mismatches (n = 14), pregnant patients (n = 3), and a transfer case (n = 1); thus 738 patients were finally enrolled.

External validation was performed at two other facilities. These two medical facilities differ in location, local population, and function as hospitals. The first external validation included 191 patients with COVID-19 who were admitted and treated at Kashiwa Kousei General Hospital (No. 21005) General Hospital. The second external validation included 230 patients with COVID-19 who were admitted and treated at Eastern Chiba Medical Center (No.161) Medical Center.

This retrospective multi-center study was approved by the Institutional Review Boards of Chiba University (No. 4074), Chiba Aoba Municipal Hospital (No. 20200301), Kashiwa Kousei General Hospital (No. 21005), and Eastern Chiba Medical Center (No.161). The study was conducted in accordance with the principles of the Declaration of Helsinki. The institutional review boards of all hospital institutions included in the present study provided ethical approval. The requirement for written informed consent was waived. To avoid any potential breach of patient confidentiality, the data were deidentified and had no linkage to the researchers.

### Clinical information

We obtained data by reviewing patient charts at the time of admission and during hospitalization. Patient background, clinical symptoms, and blood test findings were collected for clinical information. These data were collected within 24 h of the first visit or admission. Patient background included 19 items and clinical symptoms included 9 items. Blood test findings included 34 items [Supplementary Table 1(a) and (b)].

Each item was based on data obtained from at least 80% of patients from the first derivation facility. The total number of items was 62. To construct the DL model, each patient data set was normalized and any missing data were filled in using the mode method.

### Chest CT scanning

At the initial facility, the patients underwent chest CT using an 80-row CT scanner (Siemens, Erlangen, Germany). The patients were scanned from the thoracic inlet to the diaphragm during full inspiration without contrast enhancement. The CT settings were as follows: 120 kV; CT-auto exposure control; gantry rotation time, 0.5 s; and beam pitch, 0.83. All images were reconstructed using soft (I40f) and sharp reconstruction kernels (B70f) with a slice thickness of 3 mm and a reconstruction interval of 3 mm.

At the facility for the first validation, the patients underwent chest CT using a 64-row CT scanner (Siemens) and were scanned from the thoracic inlet to the diaphragm during full inspiration without contrast enhancement. The CT settings were as follows: 120 kV; CT-auto exposure control; gantry rotation time, 0.5 s; and beam pitch, 1.2. All images were reconstructed using soft (I31f) and sharp reconstruction kernels (B70f) with a slice thickness of 5 mm and a reconstruction interval of 5 mm.

At the facility for the second validation, the patients underwent chest CT using an 80-row CT scanner (Aquilion ONE; Canon Medical Systems, Otawara, Tochigi, Japan) and were scanned from the thoracic inlet to the diaphragm during full inspiration without contrast enhancement. The CT settings were as follows: 120 kV; CT-auto exposure control; gantry rotation time, 0.5 s; and beam pitch, 0.813. All images were reconstructed using soft (FC03) and sharp reconstruction kernels (FC51) with a slice thickness of 5 mm and a reconstruction interval of 5 mm. In the present study, only the soft reconstruction kernel was used for each model construction. The sharp reconstruction kernel was used for the data confirmation.

### Clinical end point

The patients were divided into two groups according to the oxygen requirements during hospitalization. In the present retrospective study, oxygen supplementation was introduced when the following conditions were confirmed: a partial pressure of arterial oxygen ≤ 60 mmHg or oxygen saturation by pulse oximetry (SpO_2_) ≤ 93% and the attending physician considered oxygen supplementation necessary, as specified by the Japanese COVID-19 guidelines [26]. An oxygen requirement was defined as 1 and no oxygen requirement was defined as 0.

The Japanese COVID-19 guideline for oxygen supply is an SpO_2_ 93% as an additional 3% error in measurement to the general standard of an SpO_2_ ≤ 90%, which reflects a PaO_2_ ≤ 60 mmHg for respiratory failure. Mild disease is an SpO_2_ ≥ 96% without respiratory symptoms or no dyspnea with cough only. No findings suggestive of pneumonia were present in any of these cases. Moderate I is an SpO_2_ > 93% but < 96% with findings of dyspnea and pneumonia and no requirement for oxygenation, but requires careful follow-up in case of deterioration. Moderate II requires oxygen administration with an SpO_2_ ≤ 93%. Critical illness requires ICU admission or ventilatory management.

### Data set

After excluding mismatch data, the data were randomly divided into training and evaluation data sets (4: 1). The total number of the data sets was 738. The number of training data sets was 591 and the number of evaluation data sets was 147. For the first external validation, the test data set was 191. For the second external validation, the data set was 230.

### DL model

**Fig. 1:**
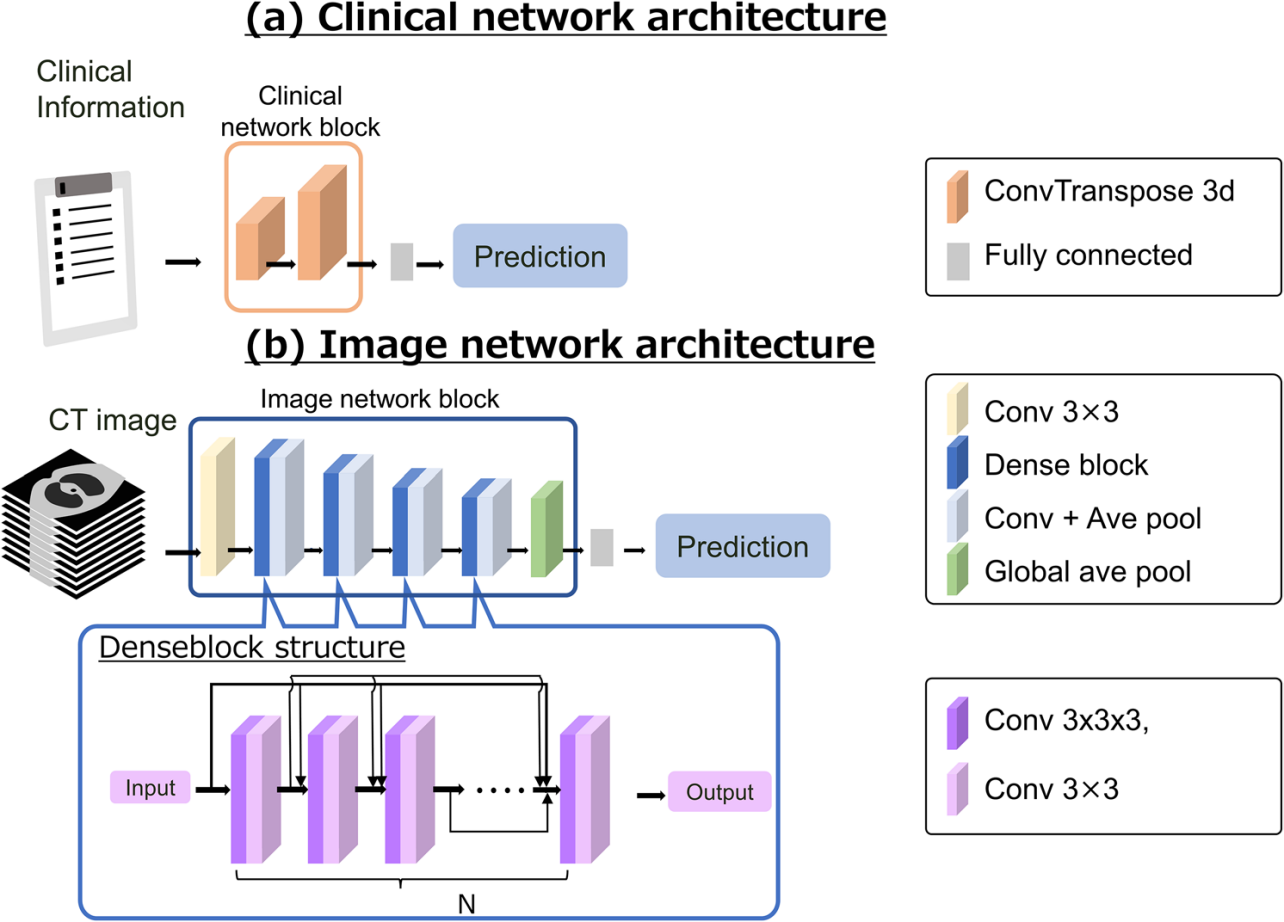
Development of the DL models. Notes: The first DL model is the clinical network architecture, which is a DL model using clinical information (**a**). The clinical information (patient background, symptoms, and blood test findings; n = 62 items) was reformed into 62 channels, convo-transposed twice, and passed through a fully connected layer to generate outputs (1 for oxygen supplementation; 0 for no oxygen supplementation). The second DL model is the image network architecture, referring to a previous report based on Densenet [22]. This model was implemented using chest CT images (**b**). After passing through the convolution layer, the model passed through three transitions: dense block, convolution layer, and average pooling. Then, after going through global average pooling, the model passed through the fully connected layer to produce the output. In each dense block, each network layer has a tightly coupled structure consisting of a 3*3 convolutional layer and a 3*3*3 convolutional layer. These layers are N-connected and have a residual structure where the outputs of each layer are added together from behind

In this study we created three DL models and compared the prediction accuracies. The first DL model was designated the clinical network architecture, which used clinical information (Fig. 1(a)). The clinical information (patient background, symptoms, and blood test findings; n = 62 items) was reformed into 62 channels, convo-transposed twice, and passed through a fully connected layer to generate outputs (1 for oxygen supplementation; 0 for no oxygen supplementation).

The second DL model was designated the image network architecture, referring to previous reports based on DenseNet [22, 27]. The image network architecture model was implemented using chest CT images (Fig. 1(b)**)**. Chest CT images were trimmed around the lungs and resized to 320 × 200 × 150 pixels. After passing through the convolution layer, the model passed through three transitions: dense block, convolution layer, and average pooling. Then, after going through global average pooling, the model passed through the fully connected layer to produce the output.

**Fig. 2:**
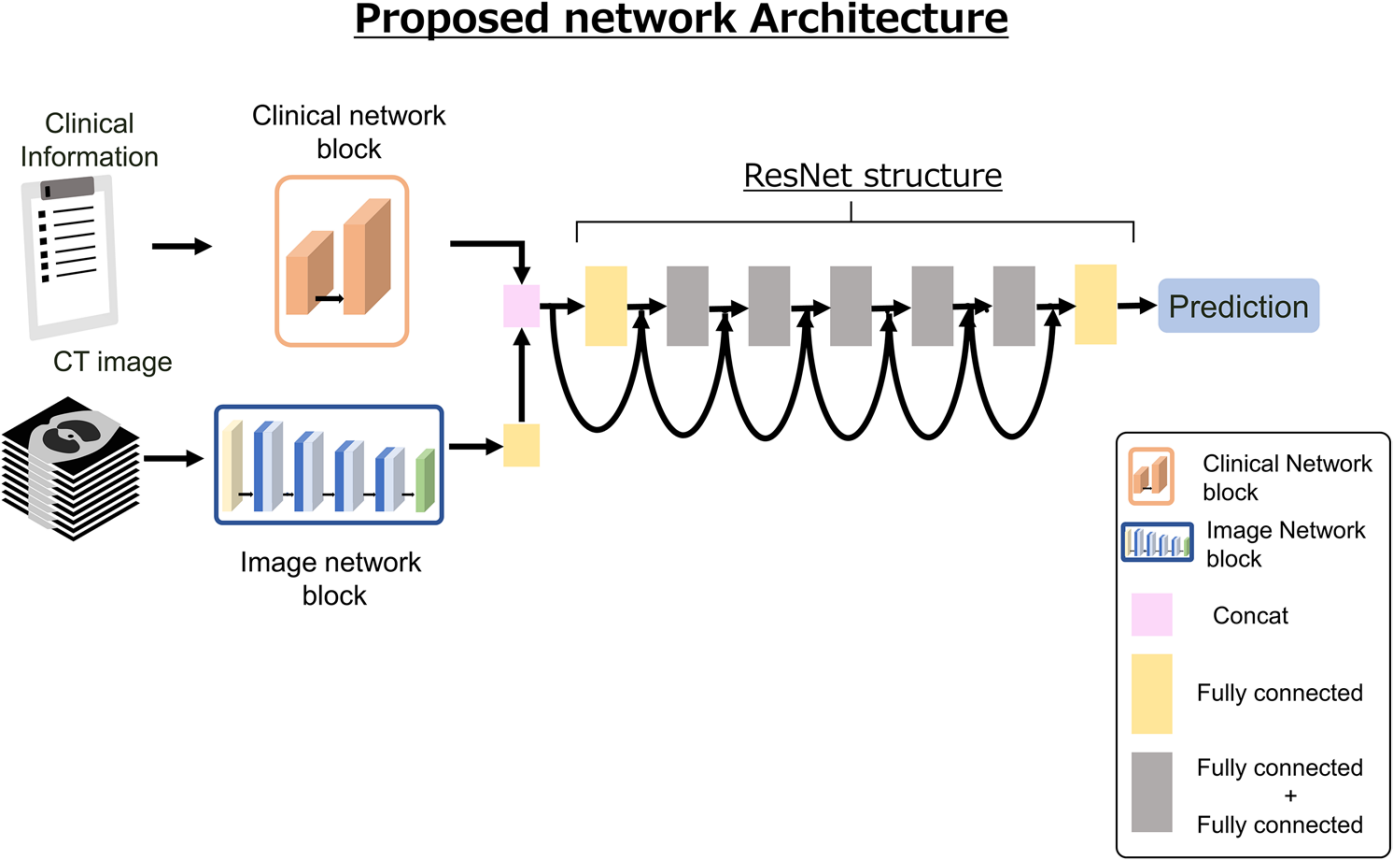
Development of the proposed DL model and proposed network architecture. Notes: The third model combined the clinical network with the image network. The DL model is the proposed network architecture. The products from the clinical network and image network were combined and passed through a fully connected layer, then through Resnet structures, and finally through a fully connected layer. Then, the final output was generated

The third model combined the clinical and image network architectures, and was designated the proposed network architecture (Fig. 2). Referring to the transfer learning method [28], the clinical network block generated by the clinical network architecture and the image network block generated by the image network architecture were fixed based on the optimal parameters, respectively. Then the clinical and image network blocks were combined and passed through ResNet [29], and finally through a fully connected layer. Only the parameters in the layers after ResNet were updated by learning. Then, the final output was generated.

The learning environment was as follows: the number of epochs was 20; the loss function was binary cross-entropy; the optimization method was Adam; and the learning rate was 0.001.

### Analysis of factors affecting the prediction of oxygen supplementation

As an additional validation, we evaluated the importance of each clinical datum. To interpret DL models, gradient-weighted class activation mapping (Grad-Cam) for the image input [30] and local interpretable model-agnostic (LIME) were used for the table data [31]. Of note, there is no general analysis method to identify the contribution of methods that combine CT images and clinical information, such as the model we created. Hence, we evaluated the importance of each item using the following formula, where M is the learned model: ${I}_{p}$ is the 3D chest CT image of patient $p \left(1\le p\le P\right)$; and ${C}_{p1},{C}_{p2}\cdots {C}_{pN}\left(N=62\right)$ are the clinical items. $M\left({I}_{p},{C}_{p1},\cdots {C}_{pn}\cdots ,{C}_{pN}\right)$ denotes the output (1 or 0) of the learned model to the input,${I}_{p},{C}_{p1},\cdots {C}_{pn}\cdots ,{C}_{pN}$.

The importance of the nth clinical item was defined as $Importanc{e}_{n}(\%)$.

$$Importanc{e}_{n}(\%)=\left(\frac{1}{P}\sum_{p}\left|M\left({I}_{p},{C}_{p1},\cdots {C}_{pn}\cdots ,{C}_{pN}\right)-M\left({I}_{p},{C}_{p1},\cdots {m}_{n},\cdots {C}_{pN}\right)\right|\right)\times 100,$$

Here, ${m}_{n}$ represents the mean of P values in terms of the nth clinical item. The importance is the absolute value of the difference between the original estimate and the estimate obtained by inputting the value of the nth clinical item of each subject fixed at mean values (${m}_{n}$) into the learned model and averaged over all patients. The importance of each of the 62 items was estimated for each of the original derivation and external evaluation data.

### Statistical analysis

The results are expressed as the mean ± standard deviation (± SD). Categorical data are expressed as a number (%). All the statistical analyses were performed using JMP Pro version 17.0 software (SAS Institute, Cary, NC, USA). Differences between the three groups were evaluated by the Kruskal–Wallis test for data and comparisons between the two groups were performed using the Steel–Dwass method. We also calculated the areas under the receiver operating curve (AUC), accuracy, sensitivity, and specificity for the prediction of oxygen supplementation during the hospitalization. Model performance was quantified by the AUC and compared using Delong’s test. A *P* value < 0.05 was considered significant.

## Results

### Characteristics of the study participants

The demographic participants are shown in Table 1. At the first facility, the average patient age was 52 years, males predominated, and the mean time interval from symptom onset to CT was 5.4 days. The chief symptom at the time of admission was fever for 86% of the patients, cough in 51%, dyspnea in 35%, fatigue in 46%, and dysgeusia or dysosmia in 26%.

**Table 1:** Demographics of the study subjects (n = 738, 191, 230)

	Original derivation (n = 738) n (%, percentage)	1st External validation (n = 191) n (%, percentage)	2nd External validation (n = 230) n (%, percentage)
Age	52 (20–99)	58 (20–97) ^†††^	62 (20–97) ^‡‡‡, ***^
Gender (male)	444 (60%)	117 (61%)	128 (56%) ^‡ **^
BMI	24.4 ± 4.6	24.8 ± 5.7	24.7 ± 4.6
Current smoker	218 (30%)	20 (10%) ^†††^	35 (15%) ^‡‡‡^
Pack-years	10.8 ± 20.3	7.5 ± 18.5^†††^	13.7 ± 23.9^***^
Alcohol consumption	381 (52%)	52 (28%) ^†††^	N.A
Symptom onset to CT (days)	5.4 ± 4.7	5.9 ± 4.2	4.7 ± 3.5 ^‡‡‡ ***^
*Co-morbidities*			
Hypertension	186 (25%)	74 (39%) ^†††^	97 (42%) ^‡‡‡^
Diabetes mellitus	96 (13%)	45 (24%) ^†††^	52 (23%) ^‡‡^
Dyslipidemia	90 (12%)	22 (12%)	44 (19%) ^‡^
Coronary disease	15 (2%)	13 (7%) ^††^	12 (5%) ^*^
Bronchial asthma	37 (5%)	10 (5%)	11 (5%)
COPD	4 (0.5%)	9 (5%) ^†††^	9 (4%) ^‡‡‡^
*Symptom*			
Fever	633 (86%)	180 (94%) ^††^	186 (81%) ^***^
Cough	373 (51%)	84 (44%)	120 (52%)
Dyspnea	253 (35%)	102 (53%) ^†††^	56 (24%) ^‡***^
Fatigue	338 (46%)	39 (20%) ^†††^	84 (37%) ^‡***^
Sore throat	201 (27%)	26 (14%) ^†††^	36 (16%) ^‡‡‡^
Diarrhea	85 (12%)	14 (7%)	27 (12%)
Nausea/vomitting	50 (7%)	9 (5%)	4 (2%)^‡‡^
Dysgeusia/dysosmia	191 (26%)	8 (4.2%) ^†††^	24 (10%) ^‡‡‡*^
None	17 (2%)	2 (1%)	9 (4%)
*Outcome*			
Oxygen supplementation	250 (34%)	130 (68%) ^†††^	97 (42%) ^***^
Oxygen supplementation at time of hospital admission	35 (5%)	126 (66%) ^†††^	62 (27%) ^‡‡‡ ***^
High-flow nasal oxygen	79 (11%)	23 (12%)	12 (5%) ^‡*^
Intubation	20 (3%)	15 (8%) ^††^	16 (7%) ^‡‡^
ECMO	2 (0.3%)	2 (1%)	4 (2%) ^‡^
Survival/death	713 (97%)/ 25 (3%)	178(93%)/13 (7%)	219(95%)/11 (5%)

Data are expressed as the mean ± standard deviation

*BMI* body mass index; *COPD* chronic obstructive pulmonary disease; *ECMO* extracorporeal membrane oxygenation; *NA* not assessed

The difference between original derivation and the first external validation

^†^p < 0.05; ††, p < 0.01; †††, p < 0.001

The difference between original derivation and the second external validation

^‡^p < 0.05; ‡‡, p < 0.01; ‡‡‡, p < 0.001

The difference between the first and second external validations

*p < 0.05; **, p < 0.01; ***, p < 0.001

A comparison between the first facility, and the first and 2nd external facilities showed differences in age and cigarette smoking status, etc. There were also differences in the proportion of subjects with hypertension, diabetes mellitus, and chronic obstructive pulmonary disease (COPD), and differences in clinical items, such as dyspnea, fatigue, sore throat and dysgeusia or dysosmia.

### Outcome of the patients

At the first facility, the number of patients receiving oxygen during hospitalization was 250 (34%), and 35 (5%) were on oxygenation at the time of admission. The number of patients with high-flow nasal canula (HFNC) therapy was 79 (11%). Twenty patients (3%) were intubated. Two patients needed extracorporeal membrane oxygenation (ECMO). Twenty-five of the total number of patients did not survive (Table 1).

Comparisons between the three groups showed that the first facility and 2 external facilities differed in the proportion of patients with oxygen supplementation (34%, 68%, and 42%, respectively). There were also differences in the proportion of patients receiving oxygen supplementation on admission (5%, 66%, and 27%, respectively). In contrast, there were no apparent differences in survival/death.

### Major blood test findings of the patients

At the first facility, the aspartate aminotransferase (AST), lactate dehydrogenase (LDH), C-reactive protein (CRP), blood glucose, and d-dimer levels were slightly elevated compared to the normal ranges (Table 2).

**Table 2:** Major blood test findings of the study subjects (n = 738, 191, 230)

Laboratory indices	Original derivation	1st External validation	2nd External validation
TP (g/dL)	7.1 ± 0.6	6.7 ± 0.6^†††^	6.8 ± 0.5^‡‡‡ *^
ALB (g/dL)	4.0 ± 0.6	3.4 ± 0.6^†††^	3.7 ± 0.6^‡‡‡***^
AG ratio	1.3 ± 0.3	1.1 ± 0.3^†††^	1.2 ± 0.3^‡‡‡***^
AST (IU/L)	40.6 ± 34.0	51.6 ± 43.9^†††^	41.1 ± 36.7^‡‡‡^
ALT (IU/L)	37.7 ± 41.2	42.6 ± 40.9	33.9 ± 28.0^*^
LDH (U/dL)	295.5 ± 146.3	368.3 ± 180.2^†††^	282.1 ± 152.7^***^
T-Bil (mg/dL)	0.69 ± 0.34	0.61 ± 0.33^†^	0.69 ± 0.32^**^
γ-GTP (IU/L)	67.6 ± 104.7	80.5 ± 98.6^†††^	56.3 ± 75.2^***^
BUN (mg/dL)	14.9 ± 7.9	16.5 ± 9.6	16.5 ± 8.2^‡‡^
Cre (mg/dL)	0.84 ± 0.34	0.93 ± 0.97	0.92 ± 0.61
UA (mg/dL)	4.6 ± 1.6	4.8 ± 1.9	N.A
eGFR (ml/min/1.73 m^2^)	77.2 ± 21.0	73.8 ± 24.9	67.1 ± 21.0^‡‡‡**^
Na (mEq/L)	137.7 ± 3.8	136.9 ± 4.5^†^	138.4 ± 3.6^‡***^
K (mEq/L)	4.0 ± 0.4	4.0 ± 0.4	4.0 ± 0.4
Cl (mEq/L)	101.1 ± 4.2	100.2 ± 4.8^†^	101.4 ± 3.7^**^
CPK (U/L)	195.4 ± 873.5	281.1 ± 1068.2^†††^	270.2 ± 1074.6^*^
CRP (mg/dL)	4.0 ± 4.8	6.8 ± 6.7^†††^	4.5 ± 5.5^‡‡‡^
GLU (mg/dL)	124.0 ± 47.0	141.0 ± 58.9^†††^	132.3 ± 47.6^‡‡‡^
WBC count (/μL)	5353 ± 2252	6071 ± 3254^†††^	5373 ± 2179^‡‡‡***^
RBC count (× 10^4^/μL)	476.8 ± 59.9	463.1 ± 71.3	463.3 ± 70.9
HGB (g/dL)	14.4 ± 1.8	14.1 ± 2.1	14.0 ± 2.1
Hct (%)	41.9 ± 4.8	41.4 ± 5.7	40.9 ± 5.8
MCV (fL)	88.1 ± 5.3	89.9 ± 5.2^††^	88.5 ± 5.4
MCH (pg)	30.2 ± 2.2	30.4 ± 2.0	30.3 ± 2.2
MCHC (%)	34.3 ± 1.3	33.9 ± 1.2^†††^	34.2 ± 1.2^*^
PLT (× 10^4^/μL)	20.4 ± 7.1	18.8 ± 7.4^††^	18.9 ± 6.2^‡^
Baso (%)	0.39 ± 0.37	0.03 ± 0.17^†††^	0.28 ± 0.27^‡‡‡***^
Eosino (%)	0.98 ± 1.62	0.40 ± 1.88^†††^	0.66 ± 1.23^‡‡‡***^
Neutro (%)	64.0 ± 18.8	74.2 ± 12.0^†††^	68.7 ± 13.8^‡‡‡***^
Lympho (%)	23.7 ± 10.6	19.3 ± 10.0^†††^	22.1 ± 10.8^*^
Mono (%)	7.3 ± 3.3	6.2 ± 3.1^†††^	7.4 ± 3.5^***^
Neutro count (/μL)	3628 ± 1928	4555 ± 2367^†††^	3847 ± 2131^‡‡‡**^
PNI	39.8 ± 6.7	39.0 ± 6.4^††^	42.0 ± 6.8^‡‡‡***^
D-dimer (ug/mL)	1.9 ± 10.9	4.2 ± 22.6	1.6 ± 4.2^‡‡‡^

Data are expressed as the mean ± standard deviation

*TP* total protein; *ALB* albumin; *AG ratio* albumin:globulin ratio; *AST* aspartate aminotransferase; *ALT* alanine aminotransferase; *LDH* lactate dehydrogenase; *T-Bil* total bilirubin; *γ-GTP* γ-glutamyltransferase; *BUN* blood urea nitrogen; *Cre* creatinine; *UA* uric acid; *eGFR* estimated glomerular filtration rate; *Na* sodium; *K* potassium; *Cl* chloride ion; *CPK* creatine phosphokinase; *CRP* C-reactive protein; *GLU* glucose; *WBC* white blood cell; *RBC* red blood cell; *HGB* hemoglobin; *Hct* hematocrit; *MCV* mean corpuscular volume; *MCH* mean corpuscular hemoglobin; *MCHC* mean corpuscular hemoglobin concentration; *PLT* platelet; *Baso* basophil; *Eosino* eosinophil; *Neutro* neutrophil; *Lympho* lymphocyte; *Mono* monocyte; *PNI* prognostic nutritional index; *NA* not assessed

The difference between the original derivation and the first external validation

^†^p < 0.05; ††, p < 0.01; †††, p < 0.001

The difference between the original derivation and the second external validation

^‡^p < 0.05; ‡‡, p < 0.01; ‡‡‡, p < 0.001

The difference between the first and second external validations

*p < 0.05; **, p < 0.01; ***, p < 0.001

Comparisons between the three groups of patients showed differences in the albumin (ALB) level, albumin:globulin ratio (AG ratio), white blood cell (WBC) count, peripheral compartment cell types (basophil (Baso), eosinophil (Eosino), neutrophil (Neutro)), and the prognostic nutritional index (PNI), etc.

### Performance of the prediction model for oxygen supplementation

**Fig. 3:**
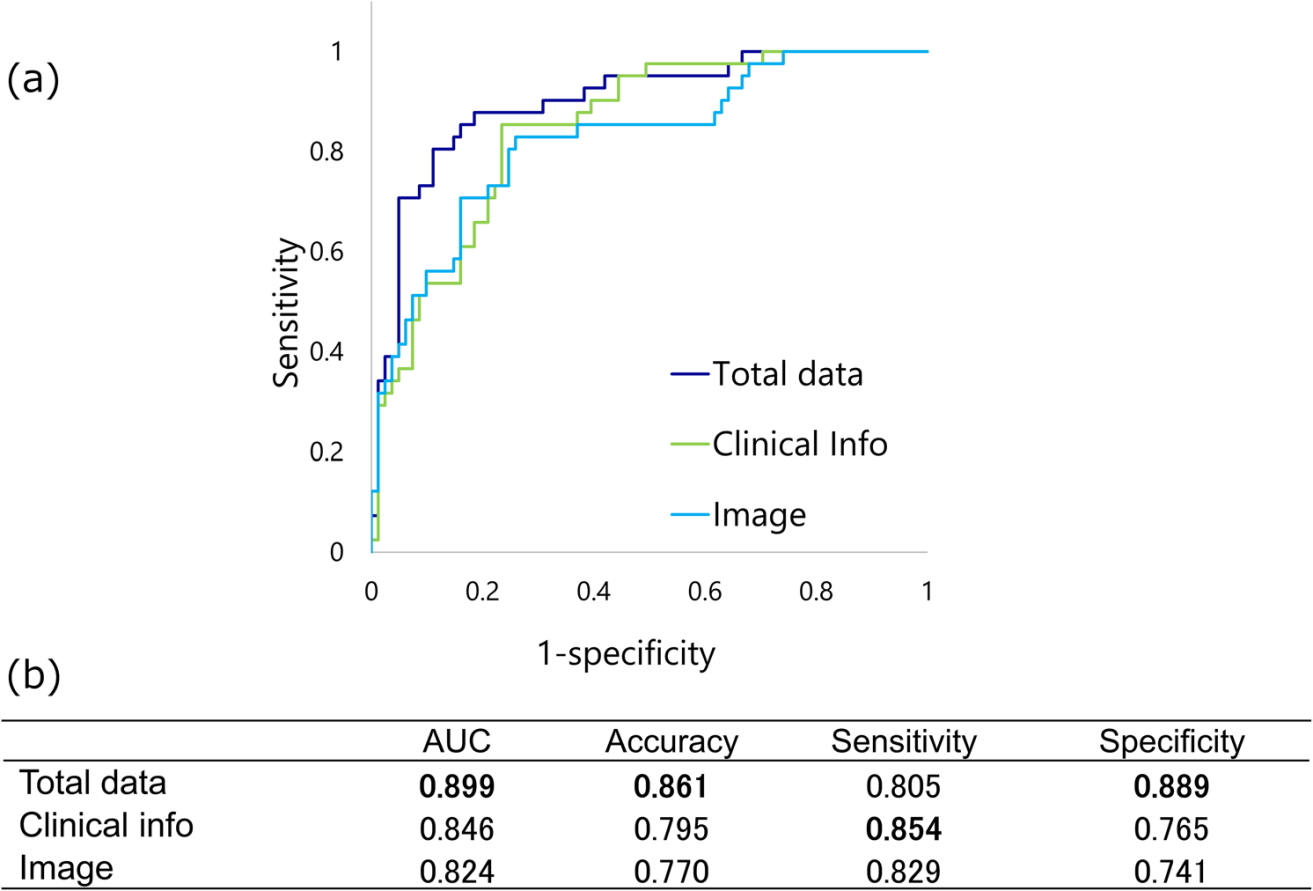
Receiver operating characteristic (ROC) curve analysis of oxygen supplementation prediction by the DL model combined with clinical information and CT images. Notes: The prediction accuracy of the model combining clinical information and CT images was higher than that of clinical information and CT images alone. Green line; Clinical information. Thin blue line; CT image. Indigo line; Total data (Clinical information + CT image)

The prediction accuracy for oxygen supplementation from the first derivation is shown in Fig. 3(a). We used the combined method to predict oxygen supplementation. In Fig. 3(b), the AUC was 0.899, the accuracy was 0.861, the sensitivity was 0.805, and the specificity was 0.889. The AUC was statistically superior for the integrated model compared to the clinical information model, and for the integrated model compared to the image model (vs. clinical information; p = 0.017 and vs. image; p = 0.007, respectively).

### Results of external facility data validations

**Fig. 4:**
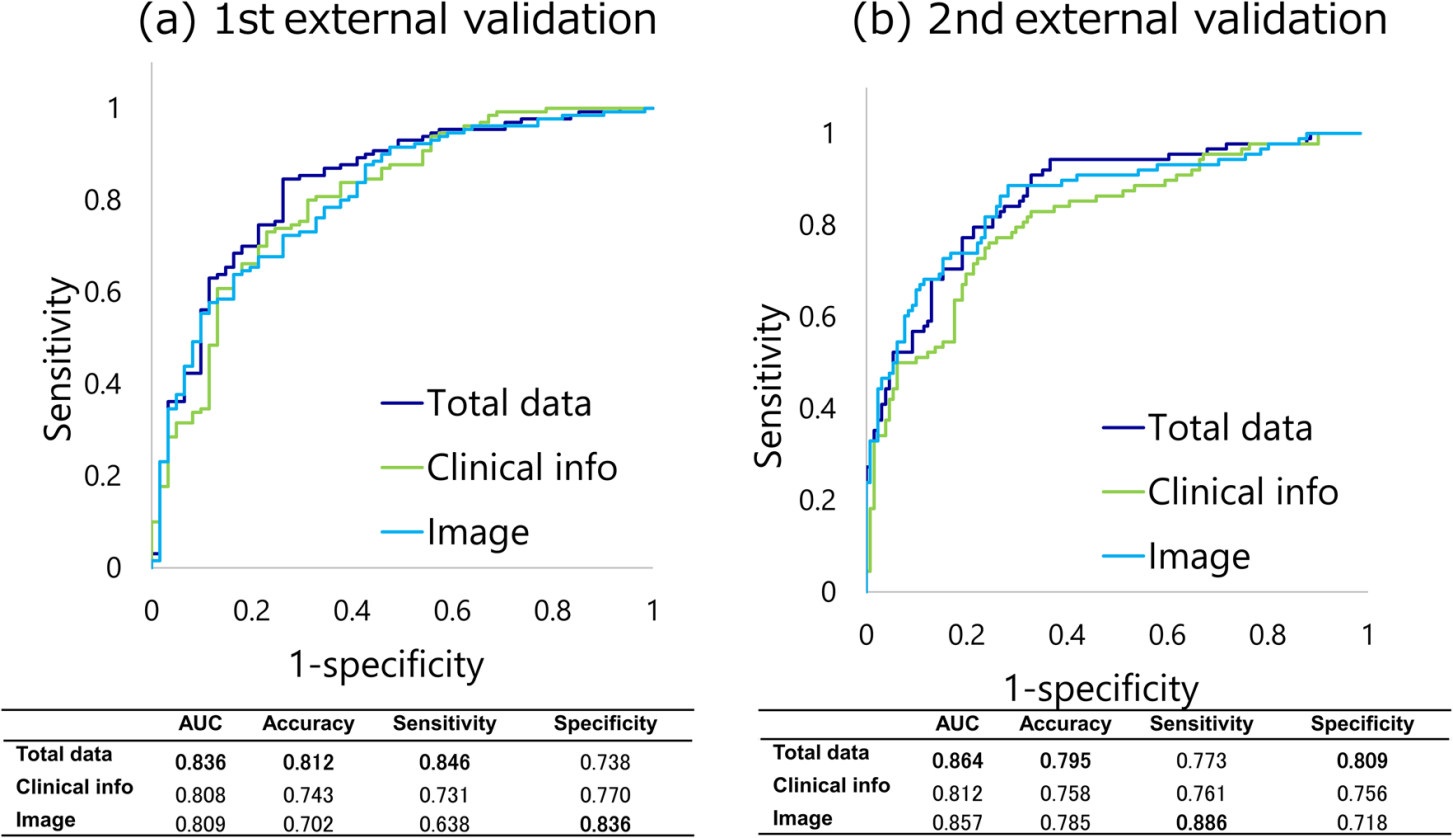
Receiver operating characteristic (ROC) curve analysis of oxygen supplementation prediction by the DL model combined with clinical information and CT images using externally-validated data. Notes: The validation results at the other two sites were > 80%. **a** and **b** First external validation. **c** and **d** Second external validation. Green line; Clinical information. Thin blue line; CT image. Indigo line; Total data (Clinical information + CT image)

To test model robustness, we tested model performance in two independent cohorts with different locations and populations, patient backgrounds, and levels of medical resources. Using the proposed method, as shown in Fig. 4**,** the AUC of the 1st external validation was 0.836 and the AUC of the 2nd external validation was 0.864. In the first external validation, there was a significant difference between the integrated and image models (p = 0.0365), while there was no significant difference between the integrated and clinical information models (p = 0.3322). In the second external validation, there was a significant difference between the integrated and clinical information models (p = 0.0026), while there was no significant difference between the integrated and image models (p = 0.7017).

### Analysis of factors influencing oxygen supplementation prediction among clinical information

**Fig. 5:**
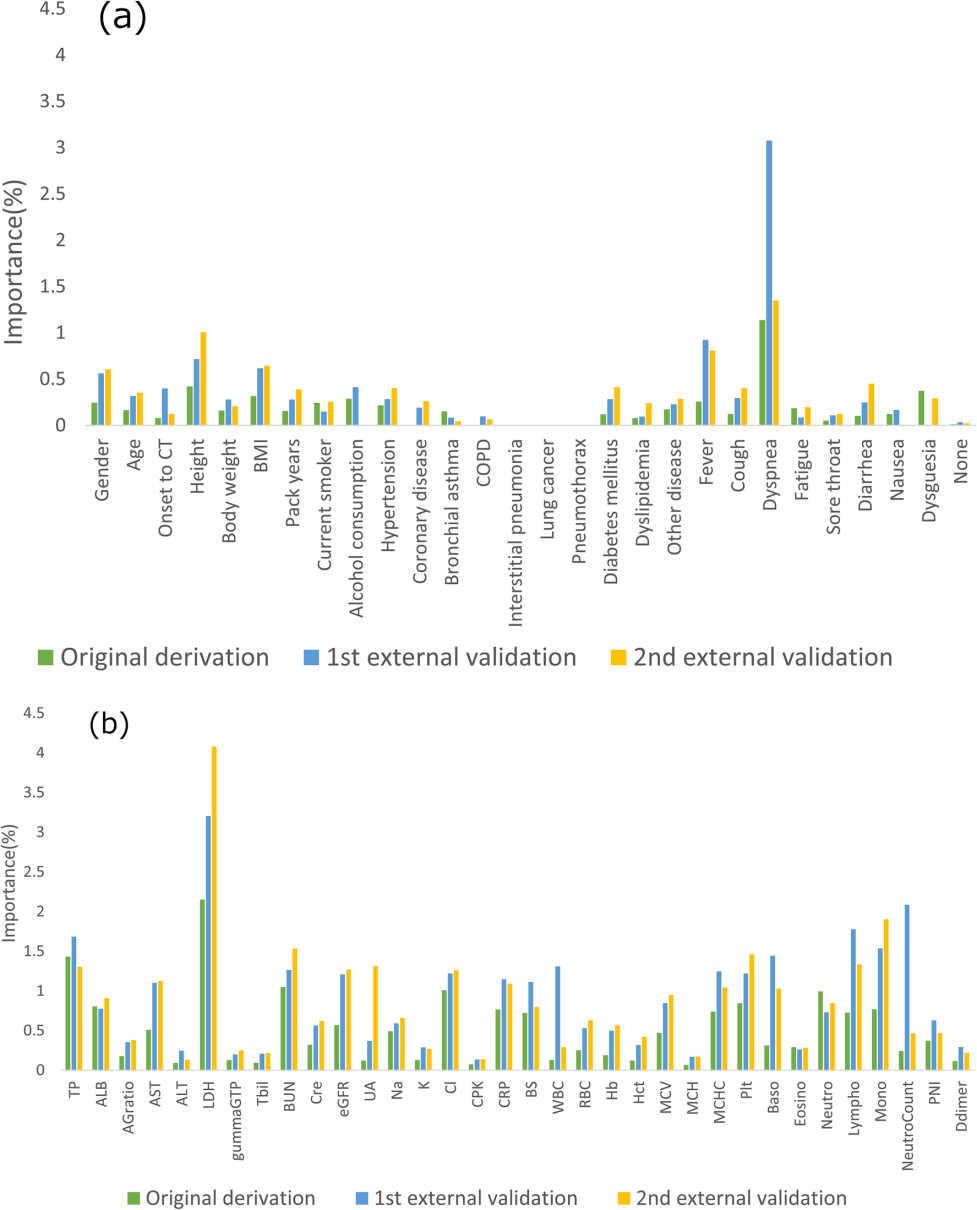
Analysis of factors affecting oxygen supplementation prediction. Notes: Using the learned parameters, the importance of each item was evaluated by the proposed formula. Dyspnea, a clinical symptom, and LDH, a blood test finding, were shown to have a strong influence on the presence of oxygen supplementation. **a** The contribution of the items among patient background and clinical symptoms. **b** The contribution of the items among blood test findings. Abbreviations: *BMI* body mass index; *COPD* chronic obstructive pulmonary disease; *TP* total protein; *ALB* albumin; *AG ratio* albumin:globulin ratio; *AST* aspartate aminotransferase; *ALT* alanine aminotransferase; *LDH* lactate dehydrogenase; *T-Bil* total bilirubin; *γ-GTP* γ-glutamyltransferase; *BUN* blood urea nitrogen; *Cre* creatinine; *UA* uric acid; *eGFR* estimated glomerular filtration rate; *Na* sodium; *K* potassium; *Cl* chloride ion; *CPK* creatine phosphokinase; *CRP* C-reactive protein; *GLU* glucose; *WBC* white blood cell; *RBC* red blood cell; *HGB* hemoglobin; *Hct* hematocrit; *MCV* mean corpuscular volume; *MCH* mean corpuscular hemoglobin; *MCHC* mean corpuscular hemoglobin concentration; *PLT* platelet; *Baso* basophil; *Eosino* eosinophil; *Neutro* neutrophil; *Lympho* lymphocyte; *Mono* monocyte; *PNI* prognostic nutritional index

Factors influencing the prediction of oxygen supplementation in clinical information are shown in Fig. 5. With respect to patient background and clinical symptoms, the presence of dyspnea was the most significant contributor (Fig. 5(a)), while LDH was the most significant blood laboratory parameter contributor (Fig. 5(b)).

## Discussion

In the present study we presented a DL prediction model for oxygen supplementation using clinical information and chest CT images in patients with COVID-19. Oxygen supplementation is one of the key factors determining the need for inpatient treatment; the AUC of the proposed model in the original facility was as high as 89.9%. Since the beginning of the pandemic, detailed integration of patient demographics and radiographic features has not been fully utilized. We have also enabled visualization of the elements on which the DL model was based.

Compared to the model built using clinical information and radiologic images separately, the model combining the two components had higher prediction accuracy. CT images and clinical information function separately as different detectors in the model. Because the patient's condition was assessed by clinical information consisting of patient background and blood test findings, and imaging findings in different ways, the combined model may have acted as a stronger detector and contributed to improved prediction accuracy. Blood test findings reflect a variety of multi-organ parameters, including systemic inflammation, the condition of various organs (such as kidney and liver function), blood glucose levels, and blood clotting markers. Blood testing findings also strongly reflect the condition of each patient at the time of the procedure and have been used for the prediction of COVID-19 severity [32, 33].

In contrast, CT imaging mainly evaluates lung findings, which have numerous imaging characteristics. It takes time for pneumonia to become established after infection, and each image has normal and abnormal areas, as well as areas of improvement and exacerbation that reflect not only the condition at the time of the procedure, but also longitudinal changes [34, 35]. Therefore, the model combining clinical information and CT images may have contributed to improvement in prediction accuracy.

The model in the present study was relatively simple, requiring only a combination of clinical findings available in an exam room and CT images in order to predict a patient's oxygen requirements. The proposed model combined DenseNet and ResNet, and integrated clinical information with CT images. Previous studies have used DL models, such as DenseNet-based models [22] and fully connected-based models [23] in COVID-19. Initially, with respect to the prediction of severe COVID-19 disease, there were limited reports from Japan, where the number of infected, severely ill, and critical cases was relatively small compared to the United States and other European countries; however, the increasing number of patients has caused a depletion of medical resources that exceeded institution capacity. According to the COVID-19 Inpatient Registry in Japan (COVIREGI-JP), the breakdown of severity among 2638 patients with COVID-19 hospitalized in the early stages of the epidemic (March-July 2020) was as follows: no oxygen (62%), oxygen (30%), and ventilator therapy in intensive care (9%) [36, 37]. A similar trend was observed in the facilities where the proposed model was derived. The presence of oxygen supplementation is one of the key factors in determining the need for hospital admission and readiness for discharge. We believe that the proposed DL model can be used to properly identify patients in need of hospitalization, which may lead to appropriate use of healthcare resources.

In the current study, we tried to visualize the factors on which the DL model was based. Since the beginning of the pandemic, gender, age, obesity, and co-morbidities (e.g., diabetes, chronic respiratory disease, cardiovascular disease, chronic kidney disease, malignancy, and immunocompromise immunity) have been reported as risk factors for severely ill patients [38]. In subsequent studies, blood tests have been reported to be predictive factors, such as CRP, erythrocyte sedimentation rate, granulocyte-lymphocyte ratio, KL-6, nutritional status, and elevated D-dimer [39–41]. In the present DL model, most of these factors were considered as clinical information inputs. As a method for evaluating the importance of the inputs to the DL estimation results, Grad-Cam for image input [42] and local interpretable model-agnostic (LIME) for table data [43] have been used in COVID-19. The analytic method for clarifying the contribution of methods that combine CT images and clinical information, such as the proposed DL model, has not been clarified. Therefore, we evaluated the importance of each item by the proposed formula using learned parameters. Dyspnea for clinical symptoms and LDH for blood tests were shown to strongly influence on the presence of oxygen supplementation. The high level of serum lactate dehydrogenase (LDH) and the presence of dyspnea have been reported to contribute to adverse outcomes in critically ill COVID-19 patients [44–46]. These two items reflect the severity and progression of SARS-CoV-2 pneumonia. The results of contributors by the proposed DL model were consistent with previous clinical reports and could provide a clear rationale for a DL model.

In this study, the prediction accuracy was reasonable at two external sites other than the first site where the DL model was constructed. The original facility and two different external facilities differed in location, local population, medical resources, and hospital functions. Specifically, in the first external validation, the integrated model improved predictive performance over the image-only model, and in the second external validation, the integrated model improved predictive performance over the clinical information-only model. We demonstrated that models based on either clinical information or images may have unstable accuracy due to the nature of the data, and suggested that integrating the two different types of data results in a more stable performance. We showed that a DL prediction model based on patient background, blood test findings, and radiologic features from one facility could be applied to other regional hospitals and outpatient clinics. To further improve the overall performance and consistency of the model, it is necessary to apply fine tuning with a small amount of data at each site. Recently, Menéndez et al. [47] created a nomogram for the severity score based on multi-center data from one surge, and showed that the results adapted to the following surges were acceptable. In the near future, our results will need to be further validated.

Numerous prognostic analyses using the DL model have been reported, especially in patients with malignant diseases [48], and many reports in COVID-19 also state that the DL model has improved the diagnostic performance of clinicians [14, 49]. The DL model proposed herein, which integrated clinical information with CT images, might be useful for the early prediction of disease severity in the next wave and for prediction of oxygen supplementation in other chronic respiratory diseases in the post-pandemic era, especially if the results can be incorporated into online entries in an examination room and electronic medical records.

This study had several limitations. First, this study was conducted as a retrospective study. Our data were obtained from a relatively small number of patients compared to general image recognition studies. Second, the internal validation showed an increase in prediction accuracy with models integrating clinical information and images. However, two external validations showed that the integrated model may not have sufficient generalization performance, with each integrated model showing only usefulness for one of the models. These results suggest that the integrated model may not be sufficiently robust to data features from different facilities. Further improvements in robustness and prediction accuracy are needed in the future, such as by increasing the number of data from other facilities in the training dataset, to improve generalization performance. Third, the duration of the study does not allow for detailed identification of the variant strain, although the type of variant strain can be estimated. In addition, patient vaccination status was not evaluated. Fourth, in the current retrospective study, it is possible that the decision to administer oxygen or not was based on careful judgement by the physician in charge especially when a patient with a chronic respiratory disease was asymptomatic or had a possible CO_2_ narcosis. Prospective studies with uniform criteria will be needed in the near future. Fifth, a longitudinal data set, including treatment for COVID-19, was not analyzed. We are conducting a corollary study to validate our model using longitudinal data, including hospitalized treatments. These preliminary results need to be confirmed in a larger multi-center longitudinal cohort.

## Conclusion

The model's prediction accuracy in combining clinical information and CT images for oxygen supplementation was high. Deep learning-based severity prediction might be helpful in clinical practice in patients with COVID-19.

### Supplementary Information

Below is the link to the electronic supplementary material.

Supplementary file1 (PDF 165 KB)

## Abbreviations

AG ratio: Albumin:globulin ratio
ALB: Albumin
ALT: Alanine aminotransferase
AST: Aspartate aminotransferase
AUC: Area under the receiver operating characteristic curve
Baso: Basophil
BMI: Body mass index
BUN: Blood urea nitrogen
Cl: Chloride ion
COPD: Chronic obstructive pulmonary disease
COVID-19: Coronavirus disease 2019
CPK: Creatine phosphokinase
Cre: Creatinine
CRP: C-reactive protein
DL: Deep learning
ECMO: Extracorporeal membrane oxygenation
eGFR: Estimated glomerular filtration rate
Eosino: Eosinophil
GLU: Glucose
Grad-Cam: Gradient-weighted class activation mapping
Hct: Hematocrit
HFNC: High-flow nasal canula
HGB: Hemoglobin
K: Potassium
KL-6: Sialylated carbohydrate antigen
LDH: Lactate dehydrogenase
LIME: Local interpretable model-agnostic explanations
Lympho: Lymphocyte
MCH: Mean corpuscular hemoglobin
MCHC: Mean corpuscular hemoglobin concentration
MCV: Mean corpuscular volume
Mono: Monocyte
Na: Sodium
NA: Not assessed
Neutro: Neutrophil
PLT: Platelet
PNI: Prognostic nutritional index
RBC: Red blood cell
RT-PCR: Real-time reverse transcriptase chain reaction
SARS-CoV-2: Severe acute respiratory syndrome coronavirus 2
T-Bil: Total bilirubin
TP: Total protein
UA: Uric acid
WBC: White blood cell
