# Supplementary material for: Prediction of oxygen supplementation by a deep-learning model integrating clinical parameters and chest CT images in COVID-19
Source: Jpn J Radiol. 2023 Jul 13;41(12):1359–72. doi: 10.1007/s11604-023-01466-3 (PMC10687147; doi:10.1007/s11604-023-01466-3)
Supplement: Supplementary file 1 — Supplementary file1 (PDF 165 KB) [file 11604_2023_1466_MOESM1_ESM.pdf]

Supplementary Table 1. Breakdown of items used as clinical information

(a) Patient background and symptoms (n = 28)

| **Variables** | |
|---|---|
| Age | Integer value |
| Gender | Categorical, Male, Female |
| Height | Integer value |
| Body weight | Continuous value |
| BMI | Continuous value |
| Current smoker | Categorical: Yes, No |
| Pack-years | Integer value |
| Alcohol consumption | Categorical: Yes, No |
| Symptom onset to CT (days) | Integer value |
| **Comorbidities** | |
| Hypertension | Categorical: Yes, No |
| Diabetes mellitus | Categorical: Yes, No |
| Dyslipidemia | Categorical: Yes, No |
| Coronary disease | Categorical: Yes, No |
| Bronchial asthma | Categorical: Yes, No |
| COPD | Categorical: Yes, No |
| Interstitial pneumonia | Categorical: Yes, No |
| Lung cancer | Categorical: Yes, No |
| Pneumothorax | Categorical: Yes, No |
| Other diseases | Categorical: Yes, No |
| **Symptoms** | |
| Fever( > 37C) | Categorical: Yes, No |
| Cough | Categorical: Yes, No |
| Dyspnea | Categorical: Yes, No |
| Fatigue | Categorical: Yes, No |
| Sore throat | Categorical: Yes, No |
| Diarrhea | Categorical: Yes, No |
| Nausea/ vomitting | Categorical: Yes, No |

| | |
|---|---|
| Dysgeusia/dysosmia | Categorical: Yes, No |
| None | Categorical: Yes, No |

(b) Blood test findings (n = 34)

| **Laboratory indices** |
|---|
| TP (g/dL) |
| ALB (g/dL) |
| AG ratio |
| AST (IU/L) |
| ALT (IU/L) |
| LDH (U/dL) |
| T-Bil (mg/dL) |
| γ-GTP (IU/L) |
| BUN (mg/dL) |
| Cre (mg/dL) |
| UA (mg/dL) |
| eGFR (ml/min/1.73 $m^2$) |
| Na (mEq/L) |
| K (mEq/L) |
| Cl (mEq/L) |
| CPK (U/L) |
| CRP (mg/dL) |
| GLU (mg/dL) |
| WBC count (/μL) |
| RBC count ($\times 10^4$/μL) |
| HGB (g/dL) |
| Hct (%) |
| MCV (fL) |
| MCH (pg) |
| MCHC (%) |
| PLT ($\times 10^4$/μL) |
| Baso (%) |
| Eosino (%) |

| |
|---|
| Neutro (%) |
| Lympho (%) |
| Mono (%) |
| Neutro count (/μL) |
| PNI |
| D-dimer (ug/mL) |

Abbreviations: TP, total protein; ALB, albumin; AG ratio, albumin:globulin ratio; AST, aspartate aminotransferase; ALT, alanine aminotransferase; LDH, lactate dehydrogenase; T-Bil, total bilirubin; γ-GTP, γ-glutamyltransferase; BUN, blood urea nitrogen;
Cre, creatinine; UA, uric acid; eGFR, estimated glomerular filtration rate;
Na, sodium; K, potassium; Cl, chloride ion; CPK, creatine phosphokinase; CRP, C-reactive protein; GLU, glucose; WBC, white blood cell; RBC, red blood cell; HGB, hemoglobin;
Hct, hematocrit; MCV, mean corpuscular volume; MCH, mean corpuscular hemoglobin;
MCHC, mean corpuscular hemoglobin concentration; PLT, platelet; Baso, basophil: Eosino, eosinophil; Neutro, neutrophil; Lympho, lymphocyte; Mono, monocyte; PNI, prognostic nutritional index
